# Comparison of the anti-diabetic and nephroprotective activities of vitamin E, metformin, and *Nigella sativa* oil on kidney in experimental diabetic rats

**DOI:** 10.22038/IJBMS.2023.68051.14876

**Published:** 2023-04

**Authors:** Hayat Ayaz, Seval Kaya, Ugur Seker, Yusuf Nergiz

**Affiliations:** 1 Dicle University, Faculty of Medicine, Department of Histology and Embryology, Diyarbakir, Turkey; 2 Istanbul Aydin University, Faculty of Medicine, Department of Histology and Embryology, Istanbul, Turkey; 3 Harran University, Faculty of Medicine, Department of Histology and Embryology, Sanliurfa, Turkey

**Keywords:** Apoptosis, Diabetes mellitus, Kidney, Metformin, *Nigella sativa*, Vitamin E

## Abstract

**Objective(s):**

In this study, we aimed to evaluate and compare the nephroprotective and possible anti-diabetic effects of vitamin E, metformin, and *Nigella sativa*.

**Materials and Methods:**

Thirty male Wistar Albino rats were randomly divided into control, experimental diabetes (DM), vitamin E + DM, Metformin + DM, and *N. sativa* + DM. For experimental diabetes induction, IP 45 mg/kg streptozotocin was administered. Rats in vitamin E + DM, Metformin + DM, and *N. sativa* + DM received 100 mg/kg vitamin E, 100 mg/kg metformin, and 2.5 ml/kg *N. sativa* oil for 56 days. After the experiment, all animals were sacrificed, and blood and kidney samples were collected.

**Results:**

The blood urea level of the DM group was significantly higher (*P*<0.01) than the control group. Urea levels in vitamin E, metformin, and *N. sativa* groups were similar to the control group (*P*>0.05) but significantly different from the DM group (*P*<0.01). Bax, caspase-3, and caspase-9 immunopositivity intensity were quite low in the control group, and similar to the *N. sativa* group (*P*>0.05). Bcl-2 immunopositivity density was highest in the *N. sativa* group, similar to the control group in terms of percentile area (*P*>0.05).

**Conclusion:**

When all three treatment methods were compared in terms of their effectiveness in alleviating DM and DN, it was determined that the most successful result was obtained with *N. sativa* oil.

## Introduction

DM is a complex disease that affects individuals with disruptions in insulin secretion and is characterized by chronic hyperglycemia (1, 2). According to the International Diabetes Federation, it is predicted that 537 million adults are suffering from DM, and this number will reach 783 million by 2045 (3). Hyperglycemia-related nephropathy, retinopathy, neuropathy, and hepatopathy are some of the important clinical outcomes and the most important reasons for mortality in DM (4). Diabetic nephropathy (DN) is one of the most common and severe microvascular complications in DM (5). Improved blood urea level is another parameter linked with renal failure in DN (6). It is also believed that long-term and uncontrolled hyperglycemic conditions, as observed in DN, may increase the accumulation of reactive oxygen species (ROS) in renal tissue that triggers apoptotic signaling and programmed cell death (7). For that reason, numerous researchers have performed experimental studies to find out the most effective, cheap, and safest treatment methods besides the only approved modality, exogenous insulin administration, in DM. Most of these experiments have focused on anti-oxidant substances due to oxidative stress being one of the most important key regulators in diabetic complications (8). 

Vitamin E is a cheap and easily reachable anti-oxidant that has the potential to scavenge ROS end products of lipid peroxidation. Vitamin E also improves kidney function parameters and prevents podocyte loss in DN (9, 10). However, the strength of vitamin E in diabetes complications has not been fully explored yet, and there are some controversial results reported in current knowledge (11-13). Although metformin is widely used in type 2 DM or type 1 DM patients with insulin resistance, recent observations indicated that it is a reliable treatment way to control type 1 DM and its complications (14).

*Nigella sativa* (NS) has been used as an alternative treatment for various diseases due to bearing anti-diabetic, anticancer, antihypertensive, and anti-inflammatory properties (15-17). For that reason, in this study, we aimed to investigate the nephroprotective effects of vitamin E, metformin, and NS in streptozocin (STZ)-induced diabetic rats comparatively.

## Materials and Methods

***Study design***

This study was carried out with the approval of the dicle University Animal Experiments Local Ethics Committee (dated 28.03.2019 and 19-04). Thirty Wistar Albino male rats, 10-12 weeks old, weighing 240-350 g, were divided into 5 groups (n=6): control, DM, DM + vitamin E, DM + Metformin, and DM + NS. The animals were housed under a 12-hr day and 12-hr light cycle at a room temperature of 24-26 °C with 55-60% humidity. During the experiment, animals were provided with standard pellet feed and tap water *ad libitum*. Animals in the control group were not exposed to any application except daily administration of 1 ml saline (*P.O.*). Experimental DM was established by dissolving a single dose of 45 mg/kg STZ (Sigma, USA) in sodium citrate solution (pH: 4.5, 0.1 M) and administering intraperitoneally to the animals in DM, DM + vitamin E, DM + Metformin and DM + NS groups (18). Blood glucose levels of animals were measured after 72 hours, and animals with ≥250 mg/dl blood glucose levels were considered experimental diabetics (19). Animals in DM + vitamin E, DM + Metformin, and DM + NS groups were administered 100 mg/kg vitamin E (20), 100 mg/kg metformin (21), 2,5 ml/kg NS oil (22) by oral gavage, respectively. At the end of the 56-day experiment, the animals were sacrificed by drawing blood from their heart.

***Measurement of biochemical parameters***

Blood samples taken from the sacrificed animals were centrifuged at 4000 rpm for 15 minutes to determine the serum urea level. Biochemistry parameters were studied with Beckman Coulter (AU5800, Germany) device using the photometric method.

***Tissue processing and staining***

The kidney tissues of the sacrificed animals were fixed in 10% formalin and embedded into paraffin blocks after routine histopathological tissue processing protocol. After that, 5 µm thick sections were obtained from paraffin blocks and stained with hematoxylin and eosin (H&E), Periodic acid–Schiff (PAS), and Masson Trichrome. For that purpose, tissue sections were deparaffinized in xylene and dehydrated through a decreasing series of alcohol, and washed in distilled water. The samples were stained with PAS (Bio Optica, lot/cat#4117) and Masson Trichrome (Bio Optica, lot/cat# 3916) with ready-to-use kits. Staining steps were performed according to the manufacturer’s instructions.

***Immunohistochemical staining***

Deparaffinized sections were passed through a decreasing series of alcohol, brought into distilled water, and washed in phosphate buffered saline (PBS). Antigen retrieval was performed in an ethylenediamine tetraacetic acid (EDTA) solution. Sections were washed in PBS and treated with 3% hydrogen peroxide solution for 20 minutes. Ultra V Block solution was applied to the sections for 8 minutes. Bax (sc7480 Santa Cruz Biotechnology, Inc. Oregon, USA dilution: 1:200), Bcl-2 (sc7382 Santa Cruz Biotechnology, Inc. Oregon, USA dilution: 1:200) caspase-3 (sc56053 Santa Cruz Biotechnology, Inc.) Oregon, USA dilution: 1:150), and caspase-9 (sc56076 Santa Cruz Biotechnology, Inc. Oregon, USA dilution: 1:250) primary antibodies were applied onto the tissue sections and incubated at +4 °C overnight. The samples were incubated with secondary antibodies and enzymes at room temperature for 15 min each. After the washing process, diaminobenzidine (DAB) was applied as a chromogen, and the reactions of the sections were monitored under a light microscope. Then, the samples were counterstained with hematoxylin and mounted with Entellan™. The obtained sections were analyzed with the Image J program. For that purpose, the ratio of DAB positive within the total tissue area was automatically measured with the software. The obtained data were analyzed statistically. 

***Statistical analysis***

Obtained datasets were evaluated statistically. For that purpose, a normality test was performed and as a result of skewness and kurtosis, it was determined to use the parametric One-Way ANOVA test. Multiple comparisons were evaluated with Tukey’s *post hoc* test and the results were shown as mean ± SD. The *P*<0.05 was considered significant.

## Results

***Biochemical results***

**Figure 1:**
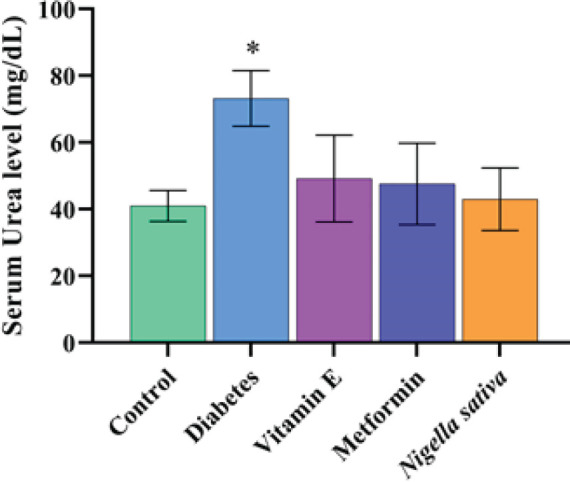
Graphical demonstration of blood urea of animals in groups. Symbols between groups indicate statistical significance *P<0.01

The lowest blood urea level was observed in the control group (41.00±4.65 mg/dl), and there was a significant difference (*P*<0.01) between the control and DM groups (73.20±8.32 mg/dl). Blood urea levels of the metformin (47.60±12.22 mg/dl) and vitamin E (49.20±13.01 mg/dl) groups were similar to the control group (*P*>0.05), and there was a significant difference (*P*<0.01) compared to DM group. It was concluded that the blood urea level of the NS (43.00±9.35 mg/dl) group was similar to the control group (*P*>0.05), but it was significantly different from the DM group (*P*<0.01). Graphical demonstrations of statistical analyses are shown in Figure 1.

***Histopathological results***

**Figure 2:**
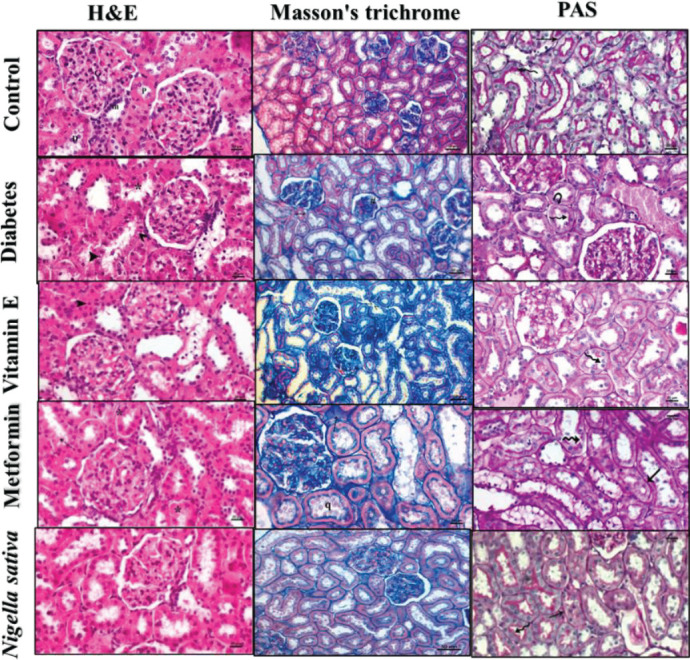
Kidney section of the groups. Macula densa (m), glomerulus (G), proximal tubule (P), distal tubule (D), tubule basement membrane (→), brush border in proximal tubules (curved arrow), necrosis in proximal convoluted tubules (*), vacuolated cytoplasm (), pycnotic nuclei in tubule epithelial cells (►), atrophic tubule (↔), atrophic glomerular (#), loss of brush border (curvy arrow), corrugation in basement membranes (), pycnosis in tubule epithelial cell nuclei (►), congestion in glomerular capillaries, perforation of tubules (><), degenerative changes (), desquamated (q) epithelial cells in the tubule lumen

Sections of the control group had normal histological structures. Atrophy and tubular necrosis were observed in the proximal convoluted tubules in the DM group. Pycnotic nuclei and cytoplasm with irregular vacuoles were detected in some tubular epithelial cells. In the sections stained with PAS, loss of the brush border, thickening, and perforation of the basement membranes were observed. We observed pycnosis in tubular epithelial cells and congestion in glomerular capillaries in the vitamin E group. Tubular atrophy was detected with irregularities in the brush margins and basement membranes in the sections stained with PAS. In the H&E stained sections of the metformin group, necrosis in tubule cells and congestion in glomerular capillaries were observed. Interstitial fibrosis was detected in Masson’s trichrome staining. Irregularities in the brush edges and basement membranes were evident in kidney sections stained with PAS. Kidney sections belonging to the NS group had a normal appearance of glomerular and tubular structures. There was no evidence of interstitial hemorrhage, tubular atrophy, and tubular necrosis. In the sections stained with PAS, glomerular basement membrane with normal histological structure, tubular basement membrane, and brush-like borders were observed (Figure 2). 

***Immunohistochemical results***

**Figure 3:**
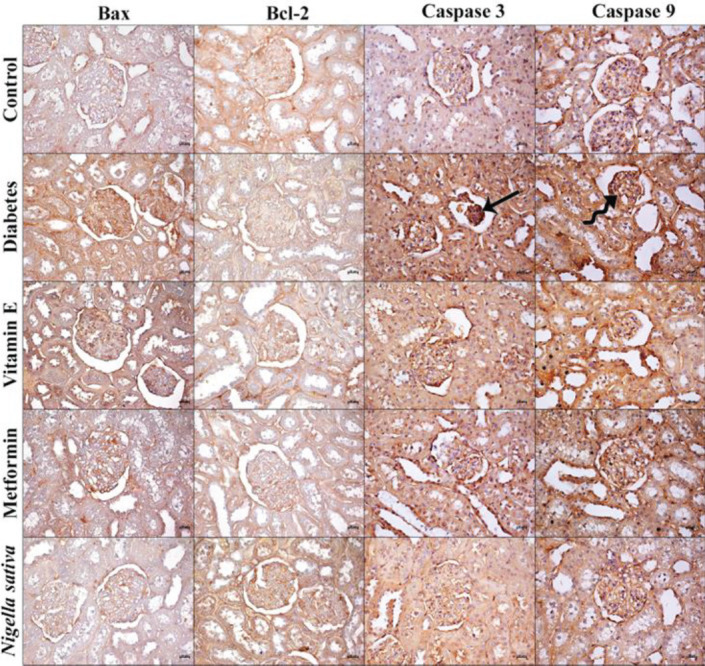
Representative micrographs of Bax, Bcl-2, caspase 3, and caspase 9 in the control, DM, vitamin E, metformin, and *Nigella sativa* groups. Increased immuodensity of caspase 3 (arrow) and caspase 9 (curved arrow) in the irregular glomerulus of diabetic animals

**Figure 4:**
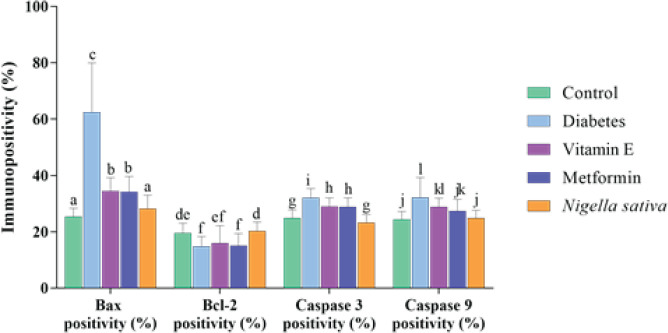
Graphical demonstration of statistical analyses. Different superscript texts on each column indicate significant differences between groups. The existence of similar characters indicates similarity. ^a-b^*P*<0.05, ^a-c^*P*<0.01, ^b-c^*P*<0.01, ^d-e^*P*<0.05, ^d-f^*P*<0.01,

^g-h^ *P*<0.01, ^g-i^*P*<0.01, ^h-i^*P*<0.05, ^j-k^*P*<0.05, ^j-l^*P*<0.01, ^k-l^*P*<0.01

Varying distribution of Bax, Bcl-2, caspase-3, and caspase-9 was observed in all groups. Although the cytoplasmic distributions of the said apoptosis-related regulatory proteins were found in the glomeruli of the kidney sections and the cells forming the structures of the tubular system, some differences were found between the groups **(**Figure 3**)**. As a result of our examination, it was determined that the immunopositivity density of Bax, caspase-3 and caspase-9 was quite low in the control group. It was determined that the expression level of all three pro-apoptotic proteins was similar to the NS group (*P*>0.05), but both the control and NS groups were significantly different (*P*<0.05) in terms of these proteins. It was observed that the Bcl-2 immunopositivity density was the highest in the NS group, and this group showed similar positivity with the control group in terms of percentile (*P*>0.05). The Bcl-2 concentration was found to be the lowest in the DM group, while the Bcl-2 level of this group was found to be similar only to the vitamin E and metformin groups (*P*>0.05). It was observed that Bax and caspase-3 levels were highest in the DM group. There was a positive difference in both Bax and caspase-3 immunopositivity intensity between the control, NS, vitamin E, and metformin groups (*P*<0.01). It was determined that the caspase-9 level was highest in the DM group, and its expression level in this group was similar to the vitamin E group (*P*>0.05). However, it was found that it was significantly different from the metformin group (*P*<0.01), and caspase-9 levels were similar (*P*>0.05) in the vitamin E and metformin groups (Table 1, Figure 4).

**Table 1:** Statistical analyses results of immunopositivity of Bax, Bcl-2, caspase 3, and caspase 9 in kidney of rats in groups

	**Baximmunopositivity (%)**	**Bcl-2immunopositivity (%)**	**Caspase 3immunopositivity (%)**	**Caspase 9immunopositivity (%)**
**Control**	25,37 ± 3,00^a^	19,47 ± 3,57^de^	24,91 ± 2,90^g^	24,36 ± 2,89^j^
**Diabetes**	62,50 ± 17,44^c^	14,86 ± 3,47^f^	32,04 ± 3,31^i^	32,28 ± 6,96^l^
**Vitamin E**	34,44 ± 4,76^b^	15,87 ± 6,28^ef^	28,94 ± 3,14^h^	28,93 ± 3,01^kl^
**Metformin**	34,16 ± 5,45^b^	15,04 ± 4,30^f^	28,88 ± 3,21^h^	27,34 ± 4,15^jk^
**Nigella sativa**	28,24 ± 4,74^a^	20,31 ± 3,14^d^	23,22 ± 2,91^g^	24,86 ± 2,83^j^

Different superscript texts between each data indicate the significant differences between the groups. The existence of similar characters indicates similarity ^a-b^*P*<0.05, ^a-c^*P*<0.01, ^b-c^*P*<0.01, ^d-e^*P*<0.05, ^d-f^*P*<0.01, ^g-h^*P*<0.01, ^g-i^*P*<0.01, ^h-i^*P*<0.05, ^j-k^*P*<0.05, ^j-l^*P*<0.01, ^k-l^*P*<0.01

## Discussion

DN is clinically characterized by albuminuria followed by a decrease in glomerular filtration rate. (23). In the experimental DM studies, it was reported that severe pathological changes occur in diabetic kidney tissue at the microscopic level (24). In a previous study, it was reported that vitamin E reduces oxidative stress due to its anti-oxidant property and has positive effects on renal function parameters, thus providing a protective effect in DN (25). Metformin is an antihyperglycemic agent frequently used in the treatment of DM (26). Metformin has also been reported to inhibit the apoptosis mechanism associated with ROS production in diabetic complications (27). It has been highlighted that NS reduces oxidative stress due to its anti-oxidant effects, provides a protective activity on renal function parameters, and reduces apoptosis in kidney tissue (28). When compared with previous studies, our results are consistent, and all of the administered compounds successfully alleviated diabetes-related kidney alterations.

In another experimental study, it was reported that the blood urea levels increased in diabetic groups compared to the control group, and this was associated with renal failure (25). On the other hand, Maheshwari *et al.* reported that serum urea levels could be controlled with the administration of metformin, regulating oxidative stress and the inflammatory cytokine release process in diabetic conditions (29). In another experimental diabetes study, the ethanolic extract of NS successfully reduced serum urea levels, hyperglycemia, and oxidative stress (24). Our results are consistent with previously published studies. During DN, tubular lining cells, renal vascular endothelial cells, and glomerular cells may suffer apoptotic cell death (30). This process is believed to be activated through the accumulation of an excessive amount of ROS that suppresses anti-apoptotic proteins by triggering the expression of pro-apoptotic proteins. In a previously published study, Sha *et al.* reported that renal cellular apoptosis is upregulated in hyperglycemic conditions due to excessive ROS accumulation, but treatment can be reached through the administration of anti-oxidant chemical substances (31). In another study, the authors reported that vitamin E has anti-apoptotic activity in experimental DM due to its anti-oxidant properties and may provide a protective effect on renal injury in DM (32). Moreover, metformin has been reported to have protective activity on various organs due to bearing not only hyperglycemic control activity but also anti-oxidant properties (33, 34). When we review the literature based on NS and DM, it is possible to comprehend that herbal treatment with NS could have promising results due to the anti-hyperglycemic and anti-oxidant effects of this plant extract (28). Until today, numerous studies are performed to explore the anti-hyperglycemic and anti-diabetic strenght of NS. In one of these, the anti-diabetic potential of NS was linked with its effective activity on increased translocation of glucose transporter type 4 (GLUT4) in skeletal muscle, storage of free glucose from the blood, and a direct enhancement of insulin sensitivity (35). Another study stated that NS decreased intestinal glucose absorption by inhibiting sodium-glucose linked transporter 1 (SGLT1) (36). Although NS was reported with its multifunctional activity on diabetes-related complications, some studies indicated that excessive anti-oxidants such as thymoquinone, carvacrol, t-anethole, 4-terpineo in NS also support treatment in DM (37). Although vitamin E does not have an insulin sensitivity-enhancing feature, research results are indicating that it can be a protective agent against the formation of DM-related nephropathy due to its intense anti-oxidant property (25, 38). Metformin inhibits glucose production by the liver by activating the AMPK signaling pathway, thereby improving insulin sensitivity and glucose uptake by striated muscles. In addition, metformin has positive effects on oxidative stress parameters in the kidney tissue of experimental diabetic rats. In a previous study, it was reported that both metformin and NS could successfully regulate oxidative stress, and our results are consistent with this study as well (17). 

## Conclusion

In our current study, in which we comparatively evaluated the anti-apoptotic and nephroprotective potentials of vitamin E, metformin, and NS, we reached findings consistent with the literature regarding that all three agents are protective at varying levels. However, as a result of our comparative analysis, we obtained strong data that the most effective nephroprotective and anti-apoptotic properties can be obtained with NS. Our findings were obtained as a result of experimental research, and we think that more research is needed to investigate the clinical usability and anti-hyperglycemic, anti-diabetic, anti-apoptotic, and nephroprotective properties of NS and to examine the molecular signal communications underlying these protective properties.

## Authors’ Contributions

HA, SK, UŞ, and YN designed the experiments; HA and SK performed experiments and collected data; HA, SK, UŞ, and YN discussed the results and strategy; YN Supervised, directed, and managed the study; HA, SK, UŞ, and Misexpresse YU Final approved of the version to be published.

## Ethical Consent

Our study was approved by dicle University Animal Experiments Ethics Committee with protocol number 19-04 and decision number 35582840-604.01.01-.

## Conflicts of Interest

 The authors declare no conflict of interest.
